# Emerging roles of ATG proteins and membrane lipids in autophagosome formation

**DOI:** 10.1038/s41421-020-0161-3

**Published:** 2020-05-26

**Authors:** Taki Nishimura, Sharon A. Tooze

**Affiliations:** Molecular Cell Biology of Autophagy, The Francis Crick Institute, London, NW1 1AT UK

**Keywords:** Macroautophagy, Endoplasmic reticulum

## Abstract

Autophagosome biogenesis is a dynamic membrane event, which is executed by the sequential function of autophagy-related (ATG) proteins. Upon autophagy induction, a cup-shaped membrane structure appears in the cytoplasm, then elongates sequestering cytoplasmic materials, and finally forms a closed double membrane autophagosome. However, how this complex vesicle formation event is strictly controlled and achieved is still enigmatic. Recently, there is accumulating evidence showing that some ATG proteins have the ability to directly interact with membranes, transfer lipids between membranes and regulate lipid metabolism. A novel role for various membrane lipids in autophagosome formation is also emerging. Here, we highlight past and recent key findings on the function of ATG proteins related to autophagosome biogenesis and consider how ATG proteins control this dynamic membrane formation event to organize the autophagosome by collaborating with membrane lipids.

## Introduction

**Fig. 1:**
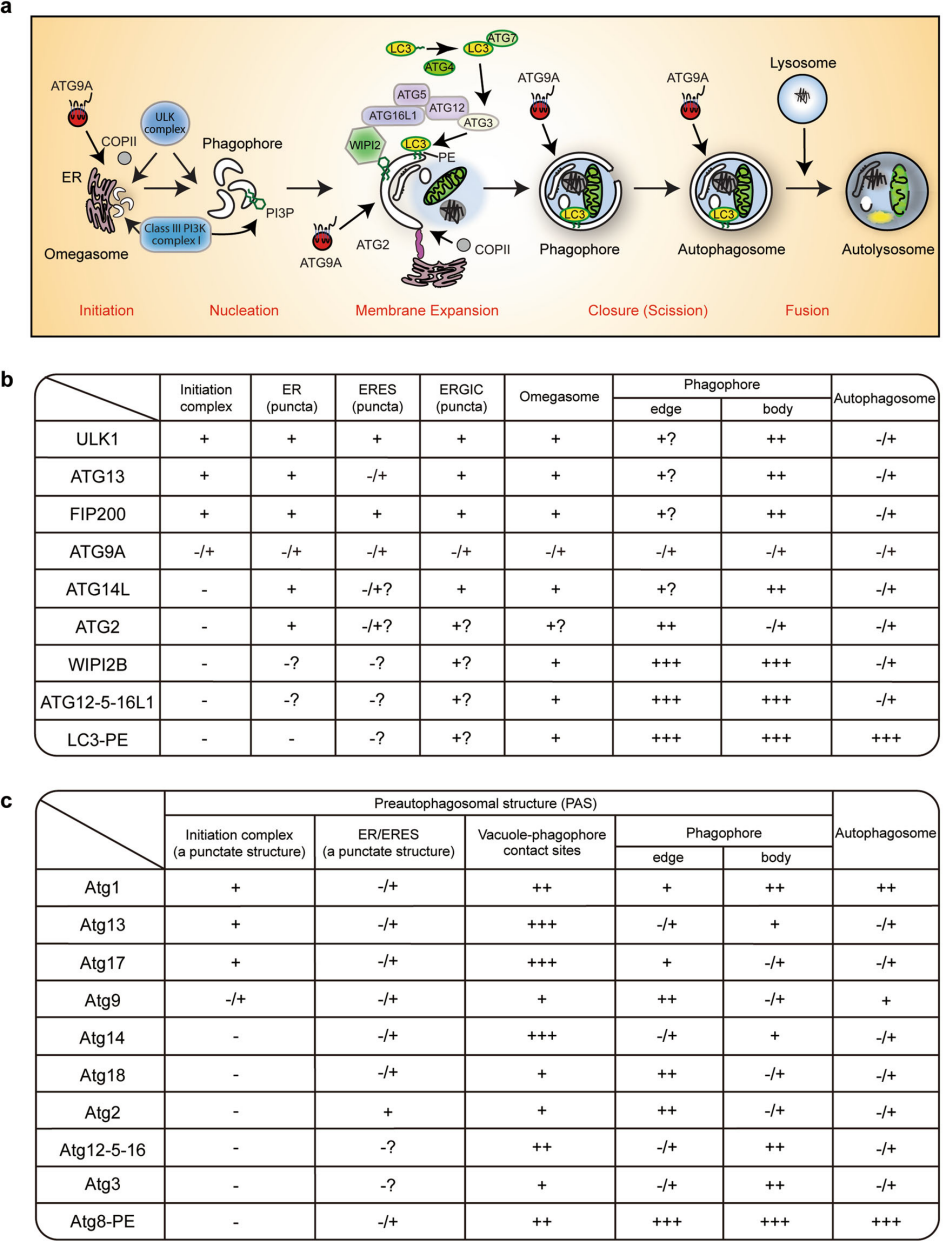
ATG/Atg proteins control dynamic membrane events during autophagosome biogenesis. **a** Autophagosome formation can be dissected into five different steps: initiation, nucleation, membrane expansion, closure, and fusion. **b**, **c** The intracellular distribution of ATG/Atg proteins under starvation-induced autophagy in mammalian (**b**)^32,34–36,48,56,58,97,127,161,172^ and yeast cells (**c**)^33,46,47,55,126,130,154,155^. Their localizations are categorized into five groups: -, not detectable; -/+, transient; +, weakly detectable; ++, easily detectable; +++, clearly detectable. Note that ATG/Atg proteins show punctate structures on the ER-related membranes rather than a typical ER-like pattern. ERES ER exit sites, ERGIC ER-Golgi intermediate compartment.

Under nutrient limited conditions cells survive by degrading their cellular contents to maintain homeostasis, energy levels, and building blocks. Macro-autophagy (hereafter, autophagy) is an intracellular degradation system that delivers cytoplasmic materials to the lysosome/vacuole. In response to autophagy induction, a cup-shaped membrane structure termed the phagophore (also known as the isolation membrane) appears in the cytoplasm. Once the phagophore elongates enough to accommodate its substrates, it closes and seals to form a double membrane structure, called an autophagosome (Fig. 1a). Initially autophagy was mainly analyzed in rat liver hepatocytes by electron microscopy^1–3^ and biochemical enzyme assays^4,5^. However, after the discovery of autophagy in the yeast *Saccharomyces cerevisiae*^6^, and the isolation of the yeast *ATG* (autophagy-related) genes^7–9^, research into autophagy has been transformed from a descriptive phenomenon into a biochemical and molecular field using model organisms, cell culture systems and in vitro reconstitution approaches. Core Atg/ATG proteins are functionally categorized into discrete units: the Atg1/ULK complex, the class III phosphatidylinositol 3-kinase (PI3K) complex, the Atg2-Atg18/WIPI4 complex, Atg9 vesicle, the Atg12 conjugation system, including ATG12–5-16L1 and WIPI2B, and the Atg8/LC3 conjugation system^10–12^. This molecular understanding of the autophagic machinery has led to the discovery of its importance in tumorigenesis^13^, mammalian development^14,15^, lipid metabolism^16^, degradation of intracellular pathogens^17^, and neurodegeneration^18,19^. In recent years, diverse physiological and pathological roles of autophagy have been uncovered^20^.

Autophagosome formation is driven by the ATG proteins, but during this dynamic membrane remodeling lipids are major constituents of autophagic membranes. Although the lipid composition of autophagosomes remains obscure, phosphatidylinositol 3-phosphate (PI3P) and phosphatidylethanolamine (PE) are crucial for autophagosome formation. The role of PI3P in autophagy was guided by a study showing the inhibitory activity of 3-methyladenine in rat hepatocytes^21^. Subsequently, it was shown that class III PI3K is a target of 3-methyladenine^22,23^. In line with this, Vps34 was identified as a PI3K in yeast^24^ and mammals^25^, and a binding partner of Vps30/Atg6^26^. Finally, the Vps34-Vps15-Vps30/Atg6-Atg14 complex was characterized as an autophagy-specific class III PI3K complex^26^. PI3P is a minor lipid, but its formation is crucial for membrane recruitment of ATG proteins and the early stage of autophagosome formation (Fig. 1a). In contrast, PE is a major phospholipid in eukaryotic cells. Under starved conditions, Atg8/LC3/GABARAP proteins are bound to autophagic membranes through covalent conjugation to PE (Fig. 1a), which is a hallmark of autophagy^27^. Although Atg8/LC3/GABARAP proteins can be also conjugated to phosphatidylserine (PS) in vitro, PE is the major target of Atg8/LC3/GABARAP conjugation in vivo^28,29^.

Considering ATG protein recruitment to autophagic membranes is both spatially and temporally controlled, changes in the amount of PI3P and PE on autophagic membranes are unlikely to fully explain how ATG proteins execute their functions and accomplish autophagosome biogenesis. Here, we review key studies and recent findings on the molecular machinery underlying autophagosome formation, and focus on the recent literature investigating the co-operation of ATG proteins with membrane lipids during autophagy, and the emerging roles of membrane lipids in autophagosome formation.

## The ULK/Atg1 complex: an initiator of autophagosome formation

**Fig. 2:**
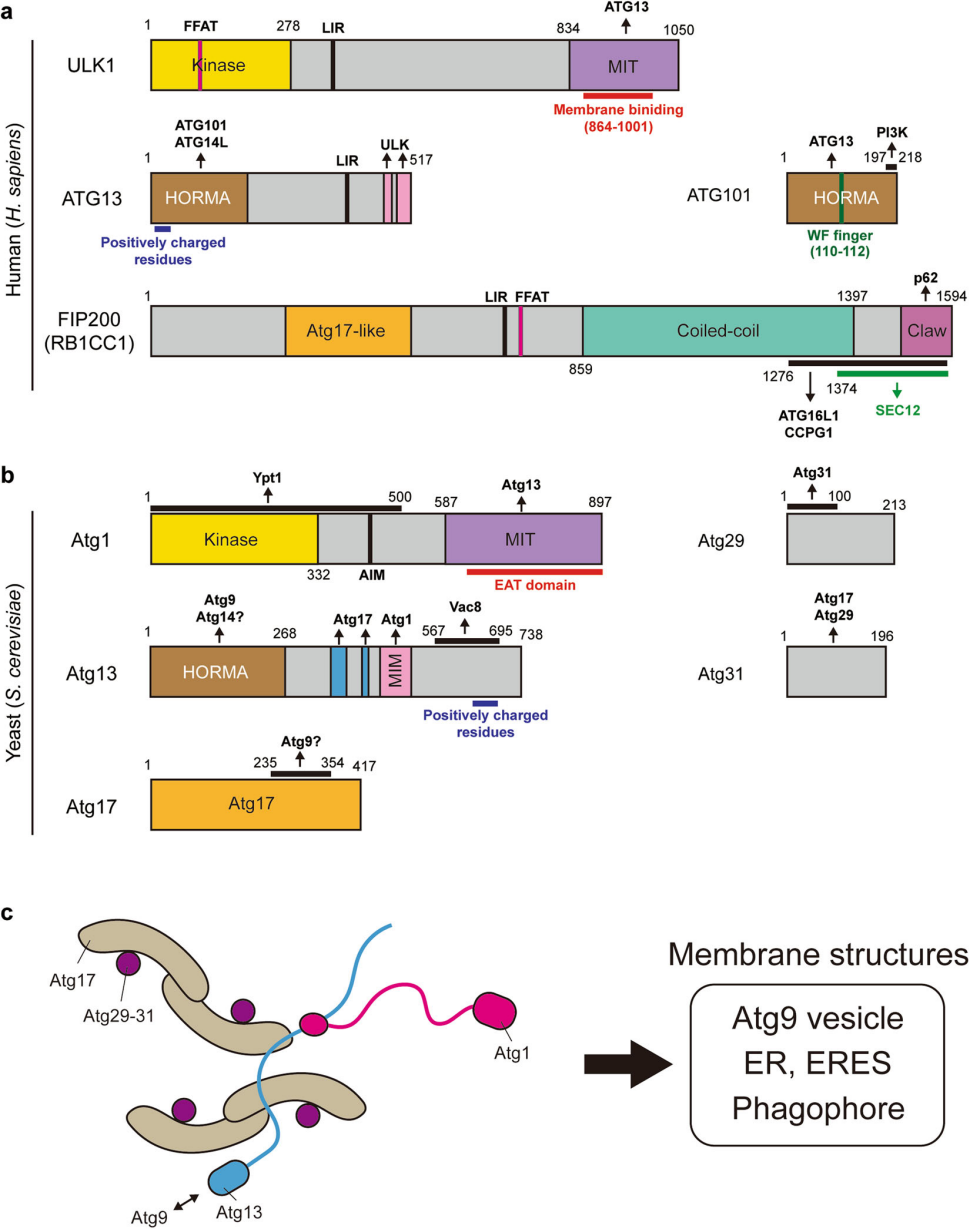
The ULK/Atg1 complex is recruited to membrane structures to initiate autophagy. **a** The domain structures of *H. sapiens* ULK complex components. **b** The domain structures of *S. cerevisiae* Atg1 complex components. **c** The proposed structure of the Atg1 complex^234^. EAT early autophagy targeting and tethering, MIT microtubule interacting and transport, MIM MIT-interacting motif, LIR LC3-interacting region, AIM Atg8 family-interacting motif, FFAT two phenylalanines in an acidic tract, HORMA Hop1/Rev7/Mad2.

The ULK/Atg1 complex is one of the earliest factors to target to membrane structures at the phagophore formation site (Fig. 1b, c). This complex composed of four components (ULK1/2, ATG13, FIP200/RB1CC1, and ATG101) in mammals (Fig. 2a), and five components (Atg1, Atg13, Atg17, Atg29, and Atg31) in yeast (Fig. 2b), is a pivotal signal transducer of autophagy. So far, the identification of ULK/Atg1 substrates has been largely confined to ones involved in autophagy^30,31^. There is accumulating evidence showing that membrane recruitment of ULK/Atg1 complex at the initiation stage does not require downstream ATG proteins. In genetic hierarchical analyses, the puncta formation of ULK/Atg1 complex is independent of other Atg complexes in both yeast and mammalian cells^32,33^. A comprehensive biochemical analysis has shown that ULK complex components are bound to membranes even in the absence of downstream ATG proteins^34^. Importantly, under starved conditions the formation of the ULK complex puncta can occur during inhibition of PI3P synthesis although the size and lifespan of puncta are reduced^34–36^. Thus, the ULK/Atg1 complex is first recruited to membrane structures independently of PI3P and downstream ATG proteins, and then its membrane localization is stabilized by PI3P^36^. Yet, which components of the ULK/Atg1 complex act as determinants for membrane recruitment is unclear.

To date, there have been several reports on the direct membrane association of the ULK/Atg1 complex components. A putative lipid-binding domain has been identified in ULK/Atg1 proteins. The C-terminus of ULK1 associates with cell membranes (Fig. 2a, red line)^37^, and in line with this, the Atg1 EAT domain (Fig. 2b, red line), a region that corresponds to the ULK1 C-terminus, binds to liposomes with a preference for small highly curved vesicles^38,39^, suggesting that the ULK/Atg1 C-terminus serves as a membrane curvature sensor. However, the Atg1 EAT domain needs to interact with Atg13 to induce autophagy^40,41^, which hinders the membrane association of Atg1 EAT domain^38^. Therefore, it is unclear if the Atg1 EAT domain in fully assembled Atg1 complex can associate with membranes. A recent paper has proposed that the Atg13-free Atg1 EAT domain is involved in later stages of autophagosome biogenesis^42^.

ATG13/Atg13 also has a putative lipid-binding region. Membrane binding of human ATG13 is regulated by a cluster of arginine/lysine residues at the N-terminus (Fig. 2a, blue line) via an electrostatic interaction with negatively charged phospholipids, such as phosphatidic acid and phosphatidylinositol phosphates (PIPs)^36^. Similarly, the C-terminus of yeast Atg13 has positively charged residues (Fig. 2b, blue line) and directly binds to liposomes containing negatively charged lipids in vitro. Thus, the lipid binding ability of Atg13 seems to be conserved from yeast to human, although the domain carrying the cluster of arginine/lysine residues is not conserved^39,43^.

Secretory pathway proteins can participate in membrane targeting of the ULK/Atg1 complex components. In yeast, a Rab GTPase Ypt1, which is essential for ER-Golgi and Golgi traffic, recruits Atg1 to the preautophagosomal structures (PAS) via an interaction with the N-terminus of Atg1 (Fig. 2b), although other Atg1 complex components, Atg13 and Atg17, are localized to the PAS independently of Ypt1^44^. The initiation step has also been spatially linked to COPII vesicles, which mediate ER-to-Golgi transport^45–48^. In mammalian cells, SEC12, the activator of COPII assembly, is associated with the FIP200 C-terminus, although this interaction is mainly required for FIP200 function in the remodeling of ER exit sites (ERES), specialized ER regions for COPII vesicle formation^48^. A recent study has revealed that ULK1 and FIP200 interact with integral ER proteins, VAP proteins (VAPA and VAPB), which can tether the ER to other cell membranes at membrane contact sites^49^. VAP proteins directly bind FFAT (two phenylalanines in an acidic tract) motif-containing proteins to the ER^50^. Consistent with this, ULK1 and FIP200 have functional FFAT motifs, suggesting that membrane association of ULK1 and FIP200 is regulated by VAP proteins. Therefore, Ypt1, SEC12, and VAPs facilitate the initiation of autophagy by recruitment of the ULK complex components. However, given that autophagic phenotypes caused by the inhibition of these interactions are partial^44,48,49^, we suggest the leading players for the recruitment of the Atg1/ULK complex are still missing (Fig. 2c).

Late stage recruitment of ULK/Atg1 complex during autophagosome formation requires LC3/GABARAP/Atg8 proteins. ULK/Atg1 complex components have a LC3/GABARAP/ATG8-interacting (LIR/AIM) motif and bind to ATG8 family proteins (Fig. 2a, b)^51–53^. These interactions have been proposed to be involved in autophagosome maturation and/or a negative feedback by degrading the ULK/Atg1 complex via autophagy.

## Atg9 vesicles: a membrane source for autophagosome formation

ATG9A/Atg9 is a multi-spanning membrane protein essential for the initiation of autophagosome formation (Fig. 1a). ATG9A/Atg9 cycles between different organelle compartments via vesicular transport pathways and delivered to the autophagosome formation site in response to induction of autophagy. In yeast Atg9 localizes to vesicular and tubular structures at the PAS under starved condition^54^. Atg9-containing vesicles, the diameter of which are 30–60 nm, are highly mobile within the cytoplasm^55^. As a small part of yeast Atg9 localizes to the autophagosomal outer membrane, it has been proposed that Atg9 vesicles become a seed membrane for phagophore formation in yeast^55^. In contrast, mammalian ATG9A is not obviously incorporated into autophagosomal membranes. Rather, ATG9A is found on clusters of vesicles and/or tubules in the vicinity of phagophores and transiently associated with the autophagosomal membranes^32,56–58^. Accordingly, it is thought that ATG9A supplies key components, such as proteins and lipids, to the autophagosomal membranes by transient association. Despite the apparent differences in the localization of ATG9A during autophagy initiation, the two models proposed in yeast and mammalian cells are not mutually exclusive.

**Fig. 3:**
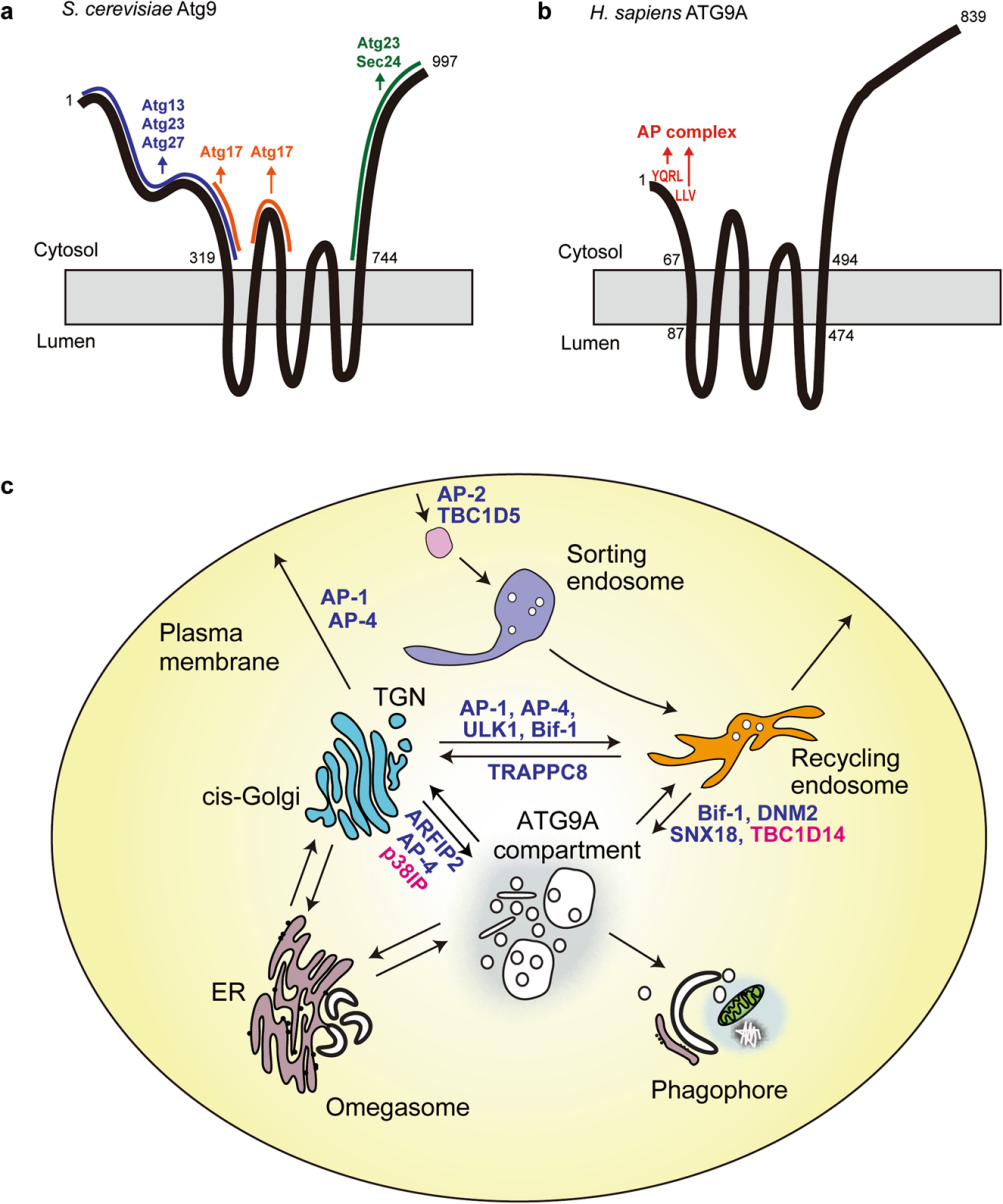
Atg9/ATG9A vesicles work as a membrane source for autophagosome formation. **a**, **b** The domain structure of *S. cerevisiae* Atg9 (**a**) and *H. sapiens* ATG9A (**b**) proteins. **c** Mammalian ATG9A cycles between different organelle compartments via vesicular transport pathways. Positive and negative regulators are shown in blue and red, respectively. AP adaptor protein, ARFIP2 arfaptin-2, SNX18 sorting nexin 18, DNM2 dynamin 2, TBC1D14 TBC1 domain family member 14, TRAPPC8 trafficking protein particle complex 8, p38IP p38-interacting protein.

Recently, the machinery sorting ATG9A/Atg9 to different locations in the cell has become increasingly clear. Atg9 distribution is partly regulated by the Atg1 complex via its physical interactions with other Atg proteins (Fig. 3a). Cytoplasmic regions of Atg9 interact with Atg17^39,59^. The N-terminus of Atg9 also binds to Atg13 HORMA domain, which facilitates recruitment of Atg9 vesicles to the PAS^60^. Atg9 is phosphorylated by Atg1 kinase, and this phosphorylation is required for recruitment of downstream factors such as Atg18 and Atg2, while it does not affect the PAS recruitment of Atg9^61^. As in yeast, the trafficking of ATG9A is dependent on the ULK complex^37,62,63^ although further analysis is needed to confirm whether ATG9A directly interacts with the ULK complex.

ATG9A/Atg9 traffic is also controlled by the vesicular transport machinery. The N-terminal tail of ATG9A contains sorting signals recognized by adaptor protein (AP) complexes and interacts with AP-1, AP-2 and AP-4 complex subunits (Fig. 3b, c)^63–65^. ATG9A distribution is altered in AP-2 knockdown^66^ and AP-4 KO cells^65,67–69^: AP-2 likely mediates transport of ATG9A from the plasma membrane and recycling endosomes, while AP-4 regulates exit from the TGN. Yeast Atg9 does not have adaptor complex recognition signals, but instead Atg23 and Atg27 function to export Atg9 into Golgi-derived vesicles^70^. Rab GTPases, GEFs (guanine nucleotide exchange factor), and GAPs (GTPase-activating protein) also control ATG9A/Atg9 trafficking, mainly between the ERGIC, Golgi, and recycling endosomes. Ypt1 is recruited to Atg9 vesicles in yeast^71,72^. Similarly, ATG9A is distributed into RAB1- and RAB11-positive compartments^73,74^. Trs85/TRAPPC8, a subunit of TRAPPIII complex (a GEF for Ypt1/RAB1) and Trs130, a subunit of TRAPPII complex (a GEF for Ypt31/Ypt32) regulate ATG9A/Atg9 traffic presumably by mediating Rab-GTP activity^73,75,76^. RabGAPs, TBC domain-containing proteins (TBC1D5 and TBC1D14), also contribute to ATG9A cycling^66,73^. TBC1D14, which has no detectable GAP activity, has been proposed to function as a effector for GTP-loaded RAB11^57^. TBC1D14 controls RAB1 activity by coupling with RAB11-GTP and the TRAPPIII complex^73^. Finally, N-BAR-containing proteins BIF-1 and sorting nexin SNX18 are involved in tubulation and fission of ATG9A-positive recycling endosomes by recruiting dynamin 2^77,78^. p38IP, an ATG9A interactor, is required for the redistribution of ATG9A in response to starvation^79^. Interestingly, phosphatidylinositol 4-phosphate (PI4P) may also regulate Atg9 traffic as a phosphatidylinositol 4-phosphate kinase Pik1 is essential for Atg9 exit from the Golgi^80^.

The essential role of ATG9A/Atg9 in autophagy remains elusive, but a recent study has revealed a close relationship between ATG9A vesicle and PI4P metabolism^81^. The BAR-domain-containing protein Arfaptin-2 and two PI4Ks (PI4KIIα and PI4KIIIβ) have been identified as components of ATG9A vesicles. ATG9A vesicles transport PI4KIIIβ to the ER membrane promoting PI4P production at the initiation site, which facilitates recruitment of the ULK complex and initiation of autophagy. In this model, Arfaptin-2 has been proposed to serve as a regulator of ATG9A vesicles by modulating ATG9A exit from the Golgi complex^81^. PI4KIIα may also be delivered by ATG9A vesicles to provide PI4P for later stages of autophagosome maturation^82^. Thus, a key role of ATG9A in autophagy may be to supply PI4P to autophagosomal membranes. Very recently, using single-particle cryo-electron microscopy the structure of *Arabidopsis thaliana* ATG9 was reported at sub-nanometer resolution^83^. Future structural studies at even higher resolution are needed to reveal some of the unresolved roles of ATG9A/Atg9.

## The class III PI3K complex I (PI3KC3-C1): a PI3P generator at the initiation site

**Fig. 4:**
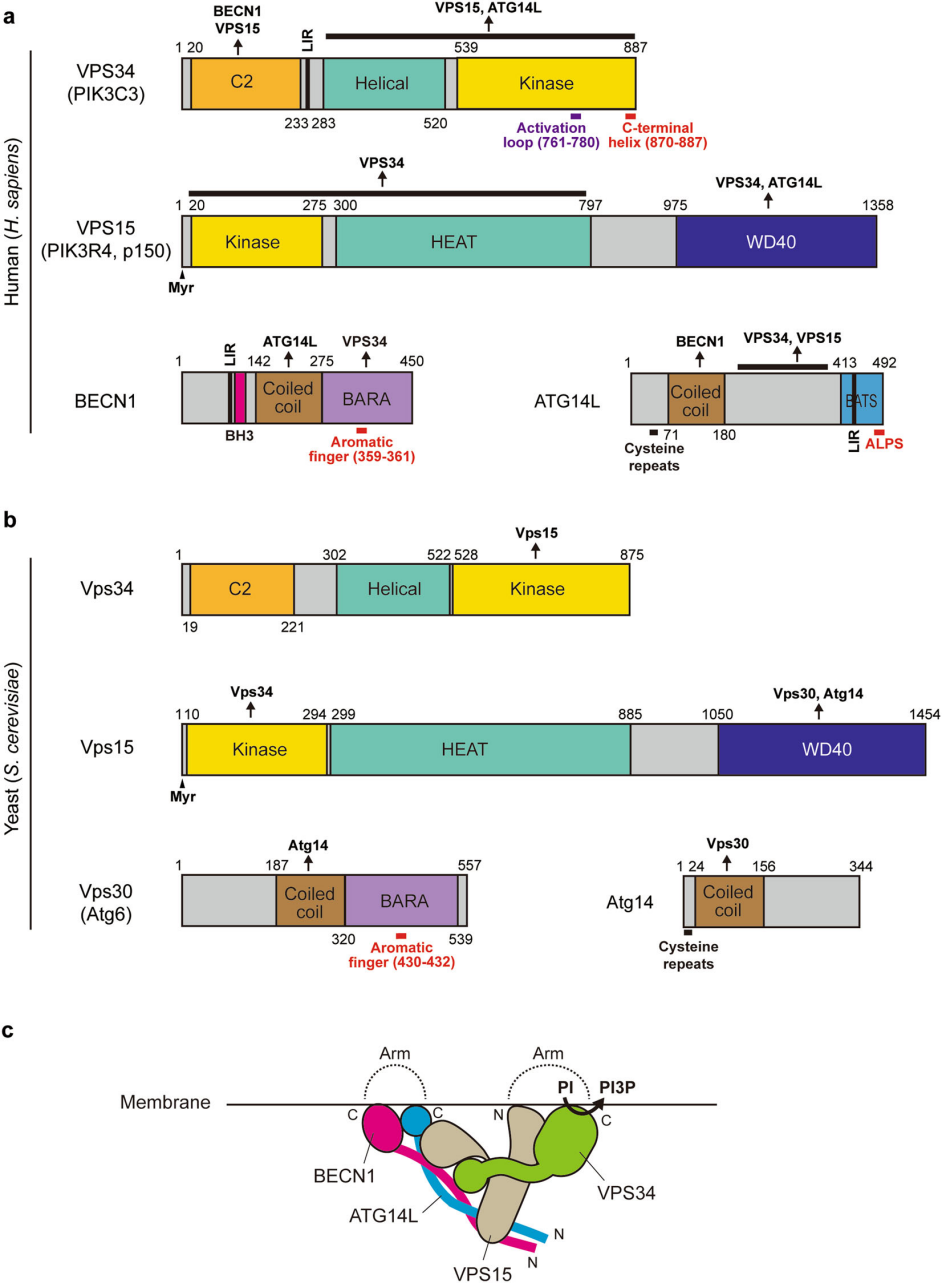
The class III PI3K complex I (PI3KC3-C1) synthesizes PI3P at the autophagy initiation site. **a**, **b** The domain structures of *H. sapiens* PI3KC3-C1 components (**a**) and *S. cerevisiae* PI3KC3-C1 components (**b**). **c** The proposed structure of mammalian PI3KC3-C1 complex. HEAT, Huntingtin, elongation factor 3, the PR65/A subunit of protein phosphatase 2A and the lipid kinase Tor; BH3 Bcl-2 homology 3, LIR LC3-interacting region, BARA β-α repeated autophagy-specific, Myr Myristoylation, BATS BAKOR and ATG14L autophagosome-targeting sequence, ALPS amphipathic lipid packing sensor.

The class III PI3K complex I (PI3KC3-C1), that is essential for the nucleation of autophagosomes, consists of Vps34/VPS34, Vps15/p150, Vps30/BECN1, and Atg14/ATG14L (Fig. 4a, b)^26,84–86^. A fifth subunit Atg38/NRBF2 facilitates the PI3KC3-C1 complex formation and further induces PI3KC3-C1 dimerization^87–92^. To generate PI3P, the PI3KC3-C1 needs to directly interact with membranes and recognize the substrate lipid, phosphatidylinositol (PI). Several studies have revealed that among the PI3KC3-C1 complex components Vps34/VPS34, Vps30/BECN1, and ATG14L have lipid-binding domains (Fig. 4a, b, red lines)^93–96^. ATG14L plays a key role in an ER-targeting of the PI3KC3-C1^97^.

Recent studies by single-particle electron microscopy and crystal structural analysis have provided many insights into how lipid-binding domains individually contribute to the membrane association of the PI3KC3-C1 complex^95,98,99^. The PI3KC3-C1 complex demonstrates a two-armed V-shaped architecture (Fig. 4c). One arm contains the helical and lipid kinase domains of Vps34/VPS34 and the Vps15/VPS15 N-terminal myristoylation site. The other arm includes the C-terminal domains of Vps30/BECN1 and Atg14/ATG14L, corresponding to BARA and BATS domains, respectively^95,98^. The crystal structure of *Drosophila melanogaster* Vps34 revealed a C-terminal loop region can interact with membranes and allow Vps34 activation^93^. The VPS34 C-terminus is regulated by acetylation at K771, which hinders the affinity of VPS34 for its substrate PI^100^. Aromatic residues in a surface loop of the BARA domain of Vps30/BECLIN1 serve as a hydrophobic finger to mediate direct association with membranes^94,95,101,102^. VPS34 catalytic site geometry is strongly influenced by the presence of VPS15, indicating that VPS15 has a central role in scaffolding complex assembly^103^. Accordingly, it is thought that the PI3KC3-C1 complex is associated with membranes via the tips of the two arms of the PI3KC3-C1 complex carrying the Vps34/VPS34 C-terminus, the myristoylated Vps15/VPS15 N-terminus, the aromatic finger in Vps30/BECN1 and the ATG14L C-terminal BATS domain (Fig. 4c). The BATS domain binds small liposomes containing PI3P or PI(4,5)P_2_ and senses membrane curvature via an amphipathic helix loop (ALPS motif, Fig. 4a)^96^. Thus, the VPS34 C-terminus determines the orientation of the PI3KC3-C1 complex, while ATG14L1 BATS domain is critical to sense membrane curvature and mediate the lipid specificity to target membranes^95,99^.

The PI3KC3-C1 complex components also contain functional LIR motifs and interact preferentially with GABARAP and GABARAPL1 (Fig. 4a). As the LIR motif in ATG14L is in close apposition to its BATS domain, these two motifs might work as a coincidence detector for specific targeting of autophagic membranes^104^.

## Atg18/WIPI proteins: a transmitter of PI3P signals

**Fig. 5:**
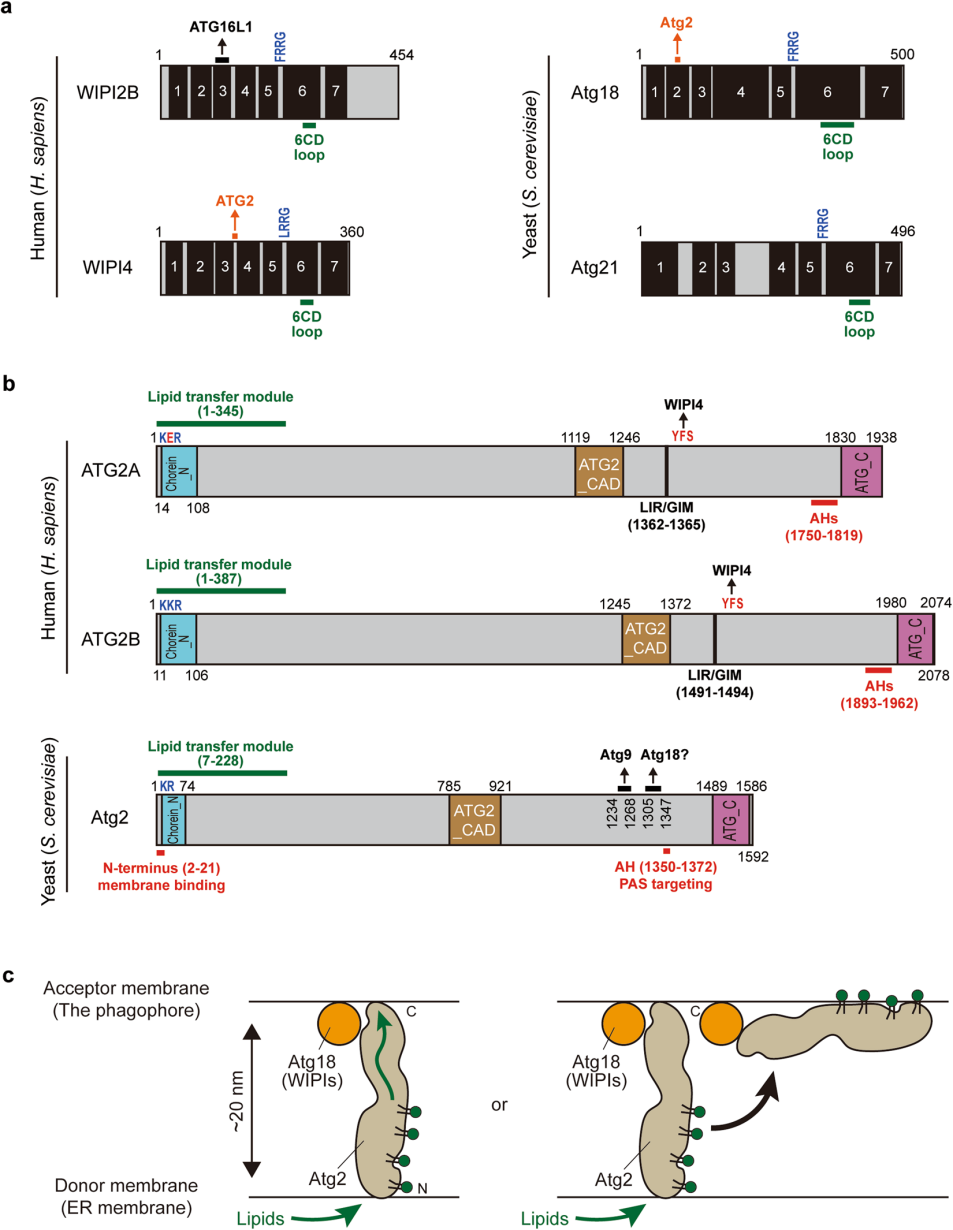
Atg2/ATG2 functions as a membrane tether and lipid transfer protein in autophagy. **a** The domain structures of WIPI2B and WIPI4 in human (left) and Atg18 and Atg21 in *S. cerevisiae* (right). **b** The domain structures of Atg2/ATG2 proteins. **c** Models of Atg2/ATG2-dependent lipid transfer. Chorein_N N-terminal region of Chorein or VPS13, Atg2_CAD autophagy-related protein 2 CAD motif, ATG_C autophagy-related protein C-terminal domain, LIR/GIM LC3/GABARAP-interacting region, AH amphipathic helix.

Atg18/Atg21/WIPI proteins belong to the PROPPINs (β-propellers that bind polyphosphoinositides) family and work as PI3P effectors in autophagy. Atg21 is likely to be restricted to yeast and function mainly in selective autophagy^105,106^. A major role of Atg18/WIPI proteins is to transmit PI3P signals to downstream ATG proteins (Fig. 5a). In yeast, Atg18 interacts with Atg2 and regulates PAS recruitment of Atg2^33,107^. In mammals, the Atg18 homolog WIPI4 interacts with ATG2 proteins^108–110^. WIPI2B most prominently functions in LC3 lipidation in mammals^111^, via its unique interaction with ATG16L1^10^. Thus, individual WIPI proteins have some preferences for their binding partners. This is the case also for *C.elegans*: the WIPI3/4 ortholog EPG-6 binds to ATG-2, while the WIPI1/2 ortholog ATG-18 does not^112^. As *C. elegans atg-18* and *epg-6* mutants show different autophagic defects, it is thought that they act at distinct steps^112^. These functional differences of Atg18/WIPI proteins in autophagy are presumably caused by differences in their binding partners^10,109,112^.

Atg18/WIPI proteins target to autophagic membranes by directly interacting with PI3P via a conserved FRRG motif (Fig. 5a, FRRG or LRRG)^113,114^. The PI3P binding of Atg18 proteins is required for full autophagic activity^107,115^. Structural analyses have revealed that Atg18 proteins fold into a seven-bladed β-propeller and contain two PI3P binding pockets at blades 5 and 6 which are composed of two arginine residues located in the conserved FRRG motif^116,117^. As Atg2/ATG2-binding and Atg16L1-binding sites are located at the opposite surface from the FRRG motif^10,108,110,118,119^, this architecture enables Atg18/WIPIs to interact with PI3P and downstream ATG proteins simultaneously.

In addition, a hydrophobic loop in blade 6 serves as a membrane anchor by inserting deeply into the lipid bilayer (Fig. 5a, 6CD loop)^117,120^. In *Komagataella phaffii* (previously known as *Pichia pastoris*), PIPs binding of PpAtg18 is modulated by phosphorylation of the loop region^121^. Of note, this loop region of yeast Atg18 can fold into an amphipathic α-helix, and is likely to be well conserved among Atg18/WIPI homologs^122^. The property of the loop might be one of main reasons why Atg18/WIPI proteins preferentially bind to smaller liposomes in vitro^120^ and localize to the edge of the phagophore in vivo^46^. Thus, direct membrane association of Atg18/WIPI proteins is controlled by two factors, membrane anchoring via the hydrophobic loop in blade 6 and PI3P-binding via the FRRG motif^117,120^. Furthermore, Atg2-Atg18 complex formation^107,119,123^ and Atg18/WIPI oligomerization^109,124^ can further stabilize the membrane association of Atg18/WIPI proteins.

## Atg2/ATG2: a membrane tether and lipid transfer protein

The precise role of Atg2/ATG2 in autophagy has recently been established. Single particle EM analyses have resolved the architecture of Atg2-Atg18 and ATG2A/B-WIPI4 complexes^108,110^. Atg2/ATG2 and Atg18/WIPI4 demonstrate a rod-shaped and a globular structure, respectively. Atg2 has membrane-binding regions at both ends of the rod structure (Fig. 5b, red lines), the length of which is about ~200 Å (Fig. 5c)^110,125,126^, and can tether small liposomes in vitro^110,126^. WIPI4 is stably attached to one end of the ATG2 rod. In a complex with WIPI4, ATG2A can tether a PI3P-containing vesicle to another PI3P-free vesicle^110^. In line with this, high-resolution microscopy analyses have shown that Atg2/ATG2 localizes to the edge of the phagophore in close apposition to the ERES in yeast^46,47^ and contact sites between ER and autophagosomal membranes in mammalian cells^127^. The N-terminal 46 residues of Atg2 localizes to the ER, and the membrane-binding region in the C-terminal region is required for the targeting of the Atg2-Atg18 complex to the PAS^126^. Furthermore, both N-terminal and C-terminal regions of Atg2 are required to restore autophagy deficiency in *atg2*△ cells. Thus, Atg2/ATG2 serves as a tether for early autophagic structures to the ER membranes by collaborating with Atg18 in yeast and WIPI4 in mammals^110,126^. Yet, the contribution of WIPI4 is limited in mammals^128,129^. Instead, a functional LIR/GIM (LC3/GABARAP-interacting motif) has been found in ATG2, which is indispensable for autophagy^129^. Atg9 also facilitates Atg2-dependent contact site formation in yeast^130^. Thus, multiple factors contribute to Atg2/ATG2-dependent tethering activity.

In addition to the tethering function of Atg2/ATG2, it possesses lipid transfer activity. Recently, the crystal structure of the N-terminus of Atg2 has been solved revealing a tubular architecture with a hydrophobic cavity that can harbor tens of glycerophospholipids at once (Fig. 5b, green lines)^131^, and can transfer phospholipids with little head group specificity using in vitro liposome assays^127,131^. The Atg2/ATG2-dependent lipid transfer depends on packing defects and negatively charged membranes^123,131^. In line with these in vitro data, the N-terminal region of Atg2/ATG2 is essential for autophagic flux in both yeast and mammalian cells^125–127,131^. Surprisingly, overexpression of the Atg2 N-terminus can restore autophagy deficiency in *ATG2A/B* DKO cells^127^, suggesting that the tethering function of Atg2/ATG2 can be rescued by overexpression of the lipid transfer domain. Given that the Vps13 N-terminus, a homologous lipid transfer domain^132^, can be substituted for the corresponding region of Atg2 during autophagy^131^, the major function of Atg2/ATG2 in autophagy is lipid transport. How Atg2/ATG2 proteins accomplish unidirectional lipid transport and grow the phagophore membranes are key issues to be solved (Fig. 5c).

## Atg16/ATG16L1: a determinant of Atg8/LC3 family lipidation sites

**Fig. 6:**
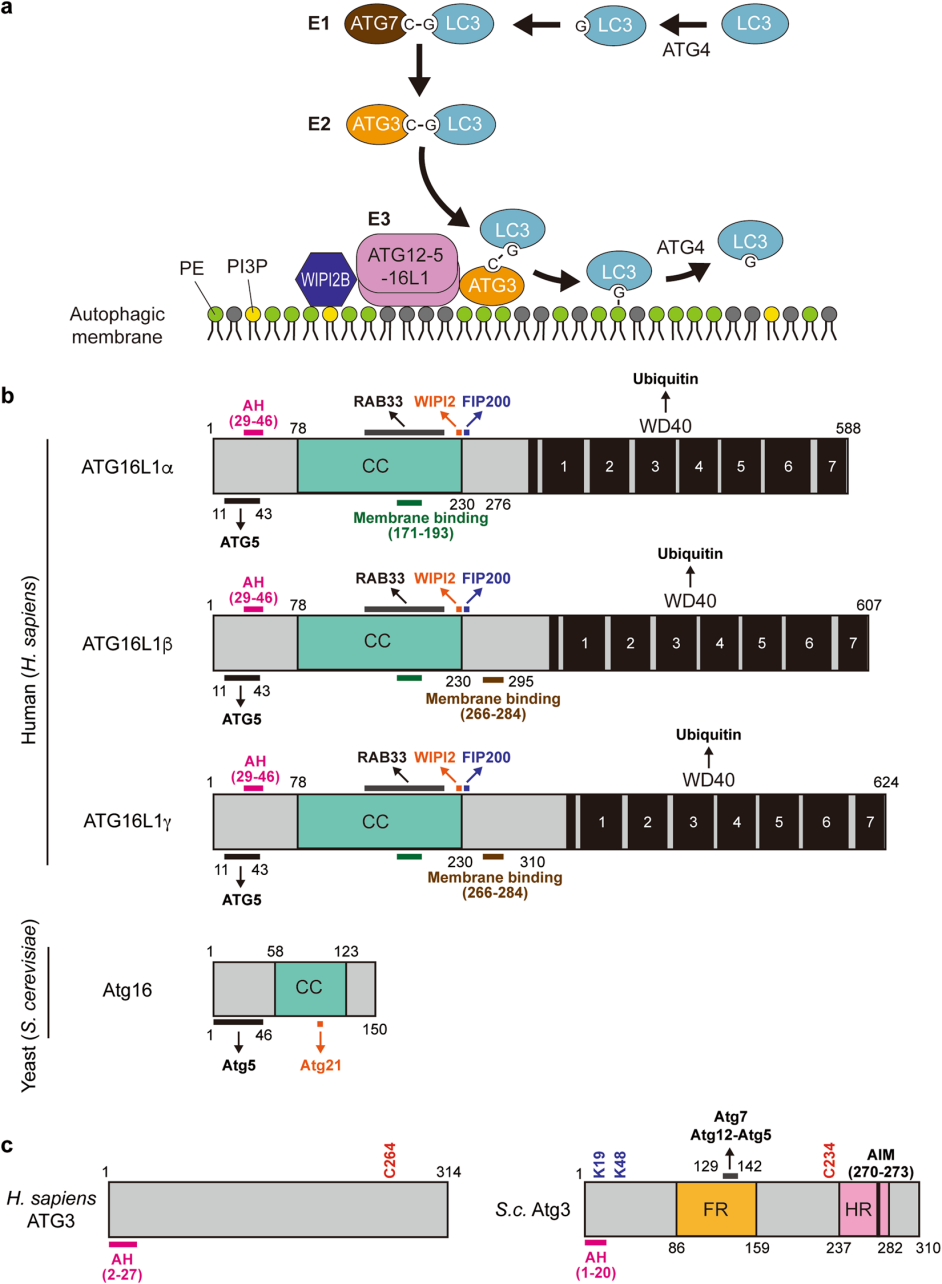
Atg12–5-16/ATG12–5-16L1 and Atg3/ATG3 catalyze lipidation of Atg8/LC3 family proteins. **a** The lipidation system of LC3. ATG4 cleaves the C-terminal residues of LC3 to expose glycine (G) residue. Then, LC3 is activated by ATG7 (E1 enzyme) and transferred to ATG3 (E2 enzyme). ATG12–5-16L1 complex facilitates the transfer of LC3 from ATG3 to PE. A PI3P-binding protein WIPI2B controls membrane recruitment of ATG12–5-16L1 complex under starvation condition. **b** The domain structures of *H. sapiens* ATG16L1 and *S. cerevisiae* Atg16 proteins. **c** The domain structures of *H. sapiens* ATG3 and *S. cerevisiae* Atg3 proteins. CC coiled-coil, AH amphipathic helix, FR flexible region, HR handle region, AIM Atg8 family-interacting motif.

The Atg12–Atg5-Atg16/ATG12–ATG5-ATG16L1 complex is composed of the Atg12–Atg5/ATG12–ATG5 conjugate and a dimeric coiled-coil protein Atg16/ATG16L1. Atg12–Atg5/ATG12–ATG5 conjugate acts as an E3-like enzyme in the Atg8/LC3-PE conjugation reaction by facilitating the transfer of Atg8/LC3s/GABARAPs from an E2-like Atg3/ATG3 to PE (Fig. 6a)^133–136^. Although Atg16/ATG16L1 is dispensable for Atg8/LC3 lipidation reactions in vitro^133,135^, Atg16 can enhance Atg8 lipidation activity against low-curvature liposomes^137^ and immobilize Atg8-PE and Atg12–Atg5 complexes on membranes in vitro^138^. In addition, Atg16/ATG16L1 has a key role in determining the site of Atg8/LC3 lipidation by controlling the targeting of the Atg12–Atg5/ATG12–ATG5 conjugate in vivo^139^.

The Atg12–Atg5-Atg16/ATG12–ATG5-ATG16L1 complex is recruited to PAS and the phagophore dependent on PI3P^140–142^. To date, several proteins have been reported as Atg16/Atg16L1-binding proteins (Fig. 6b). ATG16L1 interacts with Rab33B^143^, FIP200^144–146^ and WIPI2B^10^ via distinct amino acid residues in a region between a coiled-coil domain and a WD-repeat domain in ATG16L1. Furthermore, a WD-repeat domain in the C-terminus of mammalian ATG16L1 interacts with ubiquitin^146^. Similar interactions have been reported in yeast. Atg21, an Atg18 paralog, binds to Atg16^147^. The Atg16 complex associates with Atg1 complexes via the N-terminal region of Atg12^148^. Among these interactors, the ATG16L1-WIPI2B binding is the major PI3P-dependent interaction for membrane recruitment of ATG12–ATG5-ATG16L1 complex in starvation-induced autophagy^10^.

More recently, ATG16L1 was found to have the ability to bind lipids. ATG16L1 has three membrane-binding domains, in the N-terminal region, the coiled-coil domain (CCD) and the β-isoform-specific region. The N-terminal region, which contains an amphipathic helix (Fig. 6b, red lines), is universally required for the lipidation of LC3 family proteins, while the β-isoform-specific region (Fig. 6b, purple lines) is essential for VPS34-independent LC3 lipidation at perturbed endosomes^149^, while the CCD has an intrinsic ability to bind lipids that is also required for LC3 lipidation (Fig. 6b, green lines)^150^. In summary, the Atg16/ATG16L1 complex is recruited to the target membranes through multiple-interacting partners, including both proteins and lipids. These dependencies can be changed in response to a physiological context^149–151^.

## Atg3/ATG3: an executor of Atg8/LC3 lipidation

Atg3/ATG3 acts as the E2-like enzyme that catalyzes lipidation of Atg8/LC3 family proteins by localizing to the PAS and the phagophore (Fig. 6a)^152–155^. An amphipathic helix found in the N-terminus of Atg3 is critical for its association with membranes and Atg8/LC3 lipidation (Fig. 6c, red lines)^156–158^. This region is involved in the preferential association of Atg3/ATG3 with membranes containing high curvatures and/or conical lipids, such as PE^156^. On the other hand, lipidation of Atg8/LC3 family proteins is stimulated by acidic phospholipids in vitro^29,158^. This is probably due to the fact that membrane association of ATG3 relies on membrane charge in addition to lipid packing defects, which lead to cavities for the interaction of peripheral proteins^158^. Atg3 localization is also regulated by its interaction with Atg8 as a positive feedback to facilitate Atg8 lipidation^155^. In addition, acetylation at K19 and K48 of Atg3 significantly enhances the membrane association of Atg3 in the presence of physiological level of PE^159,160^. Overall, these multiple factors mediate Atg3 membrane targeting in vivo.

## The ER membrane: a platform for autophagosome formation

The membrane origins and sources for the autophagosome remain under debate, as several membrane sources have been proposed in mammals, including the ER^161–163^, mitochondria^164^, the PM^165^, the ERGIC^166^, the recycling endosome^167,168^, and lipid droplets (LD)^169^. Among these membrane sources it is becoming clear that the ER-related membranes are the primary membrane source and serve as a platform for autophagosome formation. In support of evidence showing the contribution of the ER membranes, omegasomes have been well characterized as an autophagy-related ER structure (ER subdomain): it is a PI3P-enriched membrane labeled by DFCP1, formed in response to starvation and dynamically connected to the ER and the phagophores^161^. Membrane contacts between the ER and phagophore have been observed by electron microscopy. Rough ER membranes are attached to both the outer and inner surfaces of cup-shaped phagophores^162,163^. Correlative light and electron microscopy (CLEM) and correlative cryo-fluorescence and cryo-soft X-ray microscopy (cryo-CLXM) have shown that omegasomes are composed of clusters of tubular/vesicular elements, part of which are continuous with the phagophores and/or the ER membranes^170,171^. These ER subdomains may also form organelle contact sites between the ER and other compartments, such as the mitochondria^172^, LDs^173^, and the PM^174^. It has been also shown that the edge regions of phagophore membranes are attached to the ERES^46–48,175^ and that COPII coat subunits are required for autophagosome formation^45,176,177^ and a COPII cargo protein is incorporated into autophagic membranes^178^, suggesting ER-derived COPII vesicles could contribute to autophagic membranes.

ATG proteins are sequentially recruited to the ER and autophagic membranes in response to autophagy induction (Fig. 1b, c). Their localization is changed depending on different stages of autophagosome formation. In mammalian cells, the ULK complex is first recruited to the ER membrane to initiate autophagy. At middle and late stages of autophagosome formation, the ULK complex also localizes to the omegasome, the phagophore and autophagosome^32,34^. ATG14L, a component of mammalian PI3KC3-C1, targets to both the ER-related membranes and phagophore to generate PI3P^96,97,172^, while the lipid transfer protein ATG2 is specifically localized to the ER and phagophore contact sites^127^. The unique localization of ATG2 presumably reflects its dual membrane targeting and tethering function. WIPI2B and ATG16L1 are mainly localized to the phagophore dependently on PI3P under starvation condition^10^. Given that it has been proposed that the ERGIC is a key membrane source for LC3 lipidation^166^, ATG12–5-16L1 and ATG3 are thought to be recruited to the ERGIC in addition to the phagophore membrane to execute LC3 lipidation. Similarly, yeast Atg proteins are also distributed to distinct compartments during autophagosome formation. Atg1 complex, Atg14, Atg12–5-16 complex, and Atg3 localize to the phagophore^46,47,154,155^, while Atg2, Atg18, and Atg9 are enriched at the edge regions of phagophore membranes, which is close apposition to the ERES^46,47,130^. On the other hand, there are some differences between yeast and mammalian cells. There are no counterparts to ERGIC and the omegasomes in yeast, but instead the phagophore is formed in the vicinity of the ERES and the vacuole^46,47,130^. Some Atg proteins such as Atg13, Atg17, and Atg14 are distributed to the vacuole and phagophore contact sites^46^. Further analyses are needed to reveal how the complex membrane targeting of Atg proteins is achieved in yeast and mammalian cells.

Membrane recruitment of the ULK complex precedes omegasome formation in mammalian cells. In high-resolution analysis using super-resolution microscopy, the initiation of autophagosome formation occurs in regions of the ER, where the ULK complex and the ATG9A vesicles are associated^58^. Interestingly, ER-localized phospholipid synthesizing enzymes, such as PI synthase and PS synthase, are enriched in close proximity to the autophagosome initiation site^34^, implying a close relationship between phospholipid synthesis and autophagosome formation. The enrichment of these enzymes might facilitate PI3P generation and ATG2-dependent lipid transfer.

## VMP1 and TMEM41B: a key regulator of ion homeostasis during autophagosome formation?

VMP1 (also known as TMEM49) is a multi-spanning membrane protein localized at the ER and required for autophagosome formation. The VMP1 gene is absent in yeast, but conserved in most higher eukaryotes. Given its ER localization, VMP1 has been thought to be a key player in an ER-related event essential for autophagosome formation. Although VMP1-GFP puncta have been observed after overexpression, this puncta formation is not essential for the autophagy function of VMP1^179,180^. Impaired VMP1 function causes not only accumulation of abnormal autophagic structures^181,182^, but also pleiotropic effects, such as protein secretion defects^183,184^, impaired lipoprotein secretion^185^, abnormal distribution of PI4P and phospholipid metabolizing enzymes^186^, accumulation of lipid droplets^179,187^, and enhanced ER-organelle contact sites^179,187^. Accordingly, VMP1 function is not limited to autophagy, and the molecular mechanism underlying the abnormalities of VMP1-deficient cells remains unclear.

A recent study has revealed a new functional link between VMP1 and a calcium pump SERCA (sarcoendoplasmic reticulum calcium transport ATPase). VMP1 interacts with SERCAs that transport cytosolic Ca^2+^ into the ER lumen and positively regulates their Ca^2+^-ATPase activity, suggesting that VMP1 coordinates multiple organelle contact sites by maintaining local Ca^2+^ levels via SERCA activity regulation^179^. More detail about VMP1 function has been provided by studies on *TMEM41B*, a newly identified autophagy-related gene in genome-wide CRISPR screens^180,188,189^. Interestingly, TMEM41B localizes to the ER and shares a similar protein structure with VMP1. As TMEM41B and VMP1 form a complex, and overexpression of VMP1 restores impaired autophagic flux in TMEM41B KO cells, it has been proposed that they may be half-transporters and function together in autophagy as a full transporter by forming a complex. More work is needed to support this exciting hypothesis and the role of VMP1 and TMEM41B in ion homeostasis during autophagosome formation.

## Emerging roles of lipid metabolism in autophagosome biogenesis

There is accumulating evidence that autophagy can be regulated by sphingolipids^190–192^. Treatment with a short-chain ceramide (C2-ceramide) is able to induce autophagy in various different cell lines, such as human cervical cancer cells, colon cancer cells, and malignant glioma cells^193–197^. Consistent with these reports, de novo synthesis of ceramide is required for the induction of autophagy in stimulated RAW264.7 macrophage cells^198^ and the mouse liver^199^, as well as in yeast^200^. The pro-autophagic effects by ceramide were proposed to be caused by upregulation of BECN1 activity and/or interfering with class I PI3K/Akt signaling pathway^194,197^, yet the molecular details remain to be elucidated.

Sphingosine 1-phosphate (S1P) is a simple lysophospholipid known to promote cell survival. Recently, the diverse roles of S1P-metabolizing enzymes in autophagy are being discovered. Overexpression of sphingosine kinase 1 (SK1), which generates S1P from sphingosine, stimulates autophagy in MCF-7 cells and primary neurons^201,202^. Depletion of S1P phosphohydrolase 1, which mediates degradation of S1P by dephosphorylation, results in the induction of autophagy, suggesting that accumulation of S1P can promote autophagy^203^. S1P not only binds to S1P receptors at the cell surface^204,205^, but also acts on intracellular membranes^202,203^, but how intracellular S1P works on autophagy remains unclear. Notably, S1P is cleaved by SGPL1 (sphingosine phosphate lyase 1) into hexadecenal and ethanolamine phosphate, which can be consumed for the synthesis of PE. Given that autophagosome formation is compromised in SGPL1-deficient brains^206^, PE generated from the S1P degradation products might play a key role in autophagy in neurons. Sphingomyelin is also involved in autophagy. Overloading of sphingomyelin affects ATG9A transport and induces accumulation of immature autophagosomes^207^. The increase of ceramide 1-phosphate (C1P) at the Golgi induced by CPTP (C1P transfer protein) knockdown also affects ATG9A distribution^208^. Neutral sphingomyelinase 2 induces autophagy by increasing ceramide levels in the Golgi^209^.

The effect of fatty acids on autophagy has drawn some attention in recent years. Saturated fatty acid palmitate induces autophagy in several cell lines^210–212^, although it has an inhibitory effect in different experimental conditions^213^. Mono-unsaturated (oleate) and ω-6 poly-unsaturated fatty acids (arachidonic acid) also activate autophagy^214–216^. Yet, it is reported that the underlying mechanisms of saturated fatty acids- and mono-unsaturated fatty acids-induced autophagy are not the same^216^. On the other hand, stearoyl-CoA desaturase, an enzyme generating mono-unsaturated fatty acids, is indispensable for efficient autophagosome formation^217–219^. Therefore, the synthetic pathway of mono-unsaturated fatty acids also contributes to autophagy. Finally, a recent study has shown that *trans-*unsaturated fatty acids inhibit autophagy induced by saturated fatty acids^220^, implying complicated connections between autophagic effects caused by different types of fatty acids. Further analyses are needed to elucidate how individual fatty acids act on autophagy.

Autophagosome formation can be affected by changes in phospholipid metabolism. PLD1 hydrolyzes phosphatidylcholine (PC) to generate phosphatidic acid (PA). In mammalian cells, PLD1 localizes to autophagosome-related structures and facilitates autophagy^221–223^. It has been proposed that its product PA plays a key role in autophagosome formation^221,224^ and/or lysosomal functions^223^. However, contrary to these findings, another study has shown that the inhibition of PLD1 results in an enhancement of autophagic flux^225^. Therefore, PLD1 might function as both positive and negative regulator of autophagy in a context-dependent manner. On the other hand, a role for lipid droplets (LDs) in autophagosome biogenesis has been reported. LDs are made of a hydrophobic core of neutral lipids and a surrounding phospholipid monolayer. In mammalian cells, a neutral lipase PNPLA5 positively regulates autophagy^169^. In line with this study, autophagy is impaired upon nitrogen starvation in LD-deficient yeast mutant^173,226^. Interestingly, a recent study has found that the autophagy deficiency in the LD-deficient yeast mutant can be improved by suppressing fatty acid synthesis or by restoring the altered phospholipid composition, indicating that LDs are not necessarily essential for autophagosome biogenesis, and that LDs play a key role in autophagy regulation by relieving metabolic stress caused by excess fatty acid synthesis and altered phospholipid composition^227^.

## Concluding remarks

Thanks to the tremendous development of novel techniques, our knowledge of the molecular machinery underlying autophagosome formation has been expanded. Of note, accumulating evidence suggest that ATG proteins are directly involved in membrane lipid dynamics and organization during autophagy. While these findings have pushed the field forward, our current knowledge of lipid composition and distribution in autophagic membranes is very limited^228^. Therefore, advanced techniques to detect and evaluate lipid distribution and changes in lipid composition in vivo need to be further developed^229,230^. In vitro reconstitution^231^, in silico simulation^232^ and theoretical analysis^233^ are also required to obtain deeper insights into the relationship between ATG proteins and membrane lipids. In summary, it will be important to reveal how ATG proteins organize membrane lipids for understanding the detailed mechanisms of autophagosome biogenesis.
